# Unveiling the role of *CtDREB1B* from safflower: enhancing plant resistance to drought and salt

**DOI:** 10.1186/s12870-026-08996-8

**Published:** 2026-05-23

**Authors:** Dandan Lu, Xiaoyu Su, Lina Wang, Lei Li, Yiwen Cao, Yongliang Yu, Chunming Li, Yao Sun, Mengfan Su, Zhengwei Tan, Huizhen Liang

**Affiliations:** 1 Institute of Chinese Herbal Medicines, Henan Academy of Agricultural Sciences, Zhengzhou, 450002 China; 2 Key Laboratory for the Protection and Utilization of Chinese Herbal Medicine Resources of Henan Province, Zhengzhou, Henan 450002 China

**Keywords:** DREB transcription factor, Safflower, Drought and salt stress, Transcriptional activation assay, Stress-responsive genes

## Abstract

**Backgrounds:**

The DREB (dehydration-responsive element binding) transcription factor family comprises key regulators that are widely used in genetic engineering to improve plant abiotic stress tolerance. However, the functional characteristics of *DREB* genes in safflower (*Carthamus tinctorius* L.) remain largely unknown.

**Results:**

In this study, a novel *DREB* gene designated *CtDREB1B,* was cloned from safflower. The full-length CDS of *CtDREB1B* was 600 bp, encoding a protein of 199 amino acids containing a conserved AP2 domain and belonging to the DREB A1 subgroup. Transient expression in maize protoplasts and tobacco leaves showed that CtDREB1B was localized to both the nucleus and cell membrane, and yeast assays verified its strong transcriptional activation activity. Expression pattern analysis revealed that *CtDREB1B* was predominantly expressed in S7 flower stage and leaves and was strongly induced by cold, drought, salt and IAA, but not by ABA and MeJA. Functional validation demonstrated that overexpression of *CtDREB1B* improved drought and salt tolerance in both yeast and transgenic *Arabidopsis thaliana*. Under drought and salt stresses, transgenic plants accumulated lower levels of reactive oxygen species (H₂O₂ and O₂⁻), exhibited higher activities of antioxidant enzymes (SOD, POD, and CAT), and showed significantly upregulated expression of core stress-responsive genes (*AtABF3*, *AtRD29A*, *AtRD29B*, *AtNCED3*, *AtCOR15A*, and *AtSOS1*).

**Conclusion:**

This study identifies a potential regulatory mechanism underlying plant drought and salt tolerance mediated by a nucleus- and membrane-localized DREB transcription factor, and provides a candidate gene resource and theoretical support for the molecular breeding of stress-tolerant crops.

**Supplementary Information:**

The online version contains supplementary material available at 10.1186/s12870-026-08996-8.

## Introduction

The AP2/ERF (APETALA2/Ethylene-Responsive Factor) superfamily is one of the largest and best-characterized plant-specific transcription factor families. AP2/ERF transcription factors play vital roles in plant growth and development, hormone signaling, pathogen defense, as well as responses to diverse biotic and abiotic stresses [1–3]. They also regulate the biosynthesis of secondary metabolites critical for plant adaptation and stress resistance [4]. Members of this superfamily typically contain one or two conserved AP2/ERF DNA-binding domain, which is composed of approximately 60 amino acids, and fold into a typical three-dimensional structure comprising 3 β-sheets and 1 α-helix [5]. Based on the number and characteristics of the AP2/ERF domains as well as overall sequence similarity, the AP2/ERF superfamily can be classified into five major subfamilies: AP2, ERF, DREB, RAV(ABI3/VP1), and Soloist [6, 7]. Among them, the ERF subfamily is further subdivided into the ERF and CBF/DREB groups [6].

DREB proteins belong to a major subfamily of the AP2/ERF superfamily, characterized by a single AP2/ERF domain, which specifically recognizes and binds to the DRE/CRT cis-elements (core sequences: ACCGAC or TACCGACAT) in the promoters of stress-responsive genes, thereby activating downstream gene expression and enhancing plant tolerance to various stresses [8, 9]. A key distinction between the DREB and ERF proteins resides in two conserved amino acid residues at positions 14 and 19 of the AP2/ERF domain: DREB proteins typically contain valine (V) and glutamic acid (E), whereas ERF proteins possess alanine (A) and aspartic acid (D) [6]. According to structural and sequence features, the DREB subfamily is further subdivided into six subgroups (A1–A6) [6].

Currently, most functional studies have focused on the A1 and A2 subgroups. Numerous studies have shown that the A1 members are predominantly involved in cold stress responses, whereas A2 members mainly function in drought, salt and heat stress tolerance [10, 11]. For example, *FaDREB1* from *Festuca arundinacea* may participate in cold stress regulation via an ABA-independent pathway [12]. Upregulation of *GhDREB1B* enhances chilling tolerance in cotton [13], and ectopic expression of *GhDREB1* in tobacco (*Nicotiana tabacum*) also increases cold tolerance [14]. Overexpression of *OsDREB1D* in *A. thaliana* enhances its tolerance to low-temperature stress [15]. In rice, transgenic plants expressing *OsDREB2A* exhibited enhanced tolerance to osmotic pressure and salinity stress under the control of an ABA-responsive promoter [16]. Similarly, overexpression of *OsDREB2A* in soybean conferred increased salt tolerance in transgenic lines [17]. Transgenic tobacco overexpressing *PgDREB2A* also displayed improved tolerance to high salt and drought stress [18]. *VrDREB2A* from cowpea was found to enhance drought and high salinity tolerance in transgenic *A. thaliana* [19]. Furthermore, transgenic *A. thaliana* and *Lotus corniculatus* roots overexpressing the *PeDREB2a* exhibited stronger tolerance to drought and high salinity stresses compared with wild-type plants [20]. Nevertheless, this functional classification is not absolute, and many DREB proteins exhibit broad and overlapping stress responsiveness, indicating more complex regulatory mechanisms than previously recognized. For instance, ectopic expression of *OsDREB1* enhances tolerance to drought, salt, and freezing stresses in both transgenic rice and *A. thaliana* [21]. The carrot *DcDREB1A* gene positively regulates drought resistance in transgenic plants by improving reactive oxygen species scavenging capacity and upregulating the expression of stress-responsive genes [22]. *MaDREB1F* confers cold and drought tolerance by binding to the promoters of *MaAOC4* and *MaACO20* and modulating the expression of these key genes involved in jasmonic acid and ethylene biosynthesis [23]. Moreover, overexpression of the lily *LlDREB1G* gene not only increases germination and survival rates but also promotes the expression of stress-related genes, thereby enhancing tolerance to drought, high temperature, and low temperature in transgenic plants [24]. Overexpression of the *EaDREB2* gene in sugarcane significantly enhances tolerance to high salt and cold stresses [25], while *AtDREB2* improves the survival rates of *A. thaliana* under cold stress [5]. *VvDREB2A* acts as a positive regulator in response to cold stress by reducing ROS accumulation, elevating raffinose family oligosaccharide (RFO) content, and promoting the expression of cold-responsive genes. Furthermore, ectopic expression of *AmDREB2C* from *Ammopiptanthus mongolicus* in *A. thaliana* enhances tolerance to freezing, heat, and drought stresses, likely by inducing the expression of *FAD* genes and facilitating C18:3 accumulation in leaves and seeds [26].

Safflower (*Carthamus tinctorius* L.) is a traditional medicinal plant in China, introduced via the Silk Road by Zhang Qian more than 2 000 years ago. As an annual herbaceous species belonging to the *Asteraceae* family, safflower is a widely utilized economic crop with applications in medicine, fabric dyeing, food coloring, ornamentation, biofuel production, and animal feed [27, 28]. Dried safflower flowers exhibit antioxidant, anticoagulant, antihypertensive, antitumor, and immunosuppressive activities, and have been widely used in traditional Chinese medicine to improve cerebral blood circulation and treat various diseases, including gynecological disorders, cerebrovascular diseases, cardiovascular diseases, hypertension, and coronary heart disease [29]. Pharmacological studies have demonstrated that the medicinal properties of safflower are attributed to the accumulation of bioactive secondary metabolites, such as phenolics and flavonoids [30, 31]. Safflower exhibits tolerance to environmental stresses including drought, salinity, extreme temperatures, and hail damage, enabling its global cultivation [32]. Nevertheless, seasonal drought and low temperature are critical factors limiting the yield and quality of safflower [33, 34]. Despite this, the functional characteristics and regulatory mechanisms of *DREB* genes involved in safflower stress adaptation remain largely unknown. Therefore, systematic identification and functional analysis of DREB transcription factors in safflower will not only deepen our understanding of the molecular regulatory network underlying abiotic stress responses, but also provide elite candidate genes and important genetic resources for the molecular breeding of stress-resistant safflower varieties.

## Materials and methods

### Plant materials and stress treatments

In this study, safflower variety Yuhonghua No. 4 was provided by Institute of Chinese Herbal Medicines, Henan Academy of Agricultural Sciences (Zhengzhou, China). Safflower plants were grown under natural conditions at the Modern Agricultural Research and Development Base of Henan Academy of Agricultural Sciences (34°55′ ~ 35°11′ N, 113°36′ ~ 114°15′ E). At the 4–6 leaf stage, young roots, stems, and leaves of 5–10 plants were collected. After budding, bracts and flowers at different developmental stages were harvested. According to the standard classification of safflower flowering stages, inflorescence development was divided into seven phases: early floral tissue formation (S1), middle floral tissue formation (S2), bud stage (S3), petal exposure stage (S4), initial flowering (S5), full flowering (S6), and early senescence (S7). Each sample had three biological replicates. After quickly freezing in liquid nitrogen, samples were stored at − 80 °C for tissue-specific expression pattern analysis of *CtDREB1B*.

To analyze the expression pattern of *CtDREB1B* under stress conditions, healthy and uniform-sized Yuhonghua No. 4 seeds were selected and sown on vermiculite, and germinated in a light incubator for 6 days. Seedlings were then cultured with Hoagland’s nutrient solution, and the solution was replaced every two days. After 2 weeks of hydroponics, seedlings with consistent growth were selected and transferred to Hoagland nutrient solution containing 20% PEG 6000, 100 mmol·L^−1^ NaCl, 100 μmol·L^−1^ ABA, 100 μmol·L^−1^ MeJA, or 100 μmol·L^−1^ IAA for treatments. For cold stress, seedlings were placed in an incubator at 4 °C. The third leaves were collected at 0, 3, 6, 12 and 24 h after treatment initiation, immediately frozen in liquid nitrogen, and stored at −80 °C. All experiments were conducted in a constant-temperature light incubator (Germplasm Resources Research Section, Institute of Chinese Medicinal Materials, Henan Academy of Agricultural Sciences) with the following settings: temperature 25 ± 2 °C, relative humidity 60% ~ 70%, light intensity 10 000 lx, and a 16 h light/8 h dark photoperiod. Each treatment included 3 biological replicates.

### Cloning and sequence analysis of *CtDREB1B*

The CDS of *CtDREB1B* was amplified using KOD One™ PCR Master Mix (TOYOBO, Osaka, Japan) with gene-specific primers (Table S1) from the cDNA of safflower. The PCR program was set as follows: pre-denaturation at 98 ℃ for 2 min; 35 cycles of denaturation at 98 ℃ for 10 s, annealing at 52 ℃ for 5 s, and extension at 68 °C for 15 s; and a final extension at 72 °C for 8 min. The total reaction volume was 20 μL, containing 10 μL KOD One™ PCR Master Mix, 2 μL cDNA, 0.6 μL each of 10 μmol·L^−1^ forward and reverse primers, and 6.8 μL sterile ddH_2_O.

PCR products were cloned into a T-vector (TaKaRa, Shiga, Japan) according to a previously reported method [35], and then sequenced by Henan Youkang Biotechnology Co., Ltd.. The amino acid sequence of CtDREB1B was deduced using DNAMAN 6.0 software. DREB amino acid sequences from various plant species were obtained from the NCBI database, and multiple sequence alignment was carried out with DNAMAN. Phylogenetic analysis was conducted using MEGA 6.0 based on the neighbor-joining algorithm, with 1 000 bootstrap replicates.

A 2 000 bp sequence upstream of the ATG start codon of *CtDREB1B* was extracted from the safflower genome database, and cis-acting element analysis was conducted using the PlantCARE online server (https://bioinformatics.psb.ugent.be/webtools/PlantCARE/HTML/) [36]. The identified cis-acting elements were visualized with TBtools.

### RNA extraction and qRT-PCR analysis

Total RNA was extracted using the FastPure® Universal Plant Total RNA Isolation Kit (RC411; Vazyme, Nanjing, China). RNA samples with an A260/A280 ratio of 1.8–2.0 and a total amount of 1 μg were used for first-strand cDNA synthesis with the HiScript® III 1 st Strand cDNA Synthesis Kit (+ gDNA wiper) (R312; Vazyme, Nanjing, China).

qRT-PCR was performed to analyze the tissue-specific expression of *CtDREB1B*, its expression patterns under stress conditions, and the expression of stress-responsive genes in *A. thaliana* under salt and drought treatments, specific primers are listed in Table S1. *Ct60S* and *AtActin* were used as the internal reference genes. Amplification reactions were performed on a QIAquant 96 2-plex Real-Time Detection System (Qiagen, Hilden, Germany). The total reaction volume was 10 μL, containing 5 μL of RealStar Fast SYBR qPCR Mix, 1.0 μL of cDNA (10 ×), 0.3 μL each of 10 μmol·L⁻^1^ forward and reverse primers, and 3.4 μL RNase-free ddH₂O. The qPCR program was as follows: pre-denaturation at 95 °C for 30 s; 40 cycles of denaturation at 95 °C for 10 s and annealing at 60 °C for 30 s. Each sample included three biological replicates and three technical replicates. Relative expression levels were calculated using the 2^⁻ΔΔCT^ method [37], and significant differences were analyzed using IBM SPSS 20 software.

### Subcellular localization of CtDREB1B

The full-length *CtDREB1B* CDS, excluding the stop codon, was amplified as described above and inserted into the pSUPER1300-EGFP vector between the HindIII and KpnI restriction enzyme sites using the ClonExpress II One Step Cloning Kit (Vazyme, Nanjing, China) following the manufacturer’s protocol. The recombinant plasmid 35S::CtDREB1B-GFP was transformed into *Agrobacterium tumefaciens* strain GV3101, and then infiltrated into the abaxial surface of tobacco (*Nicotiana benthamiana*) leaves as described previously [38].

To further confirm the subcellular localization of CtDREB, maize leaf protoplasts were isolated and transfected with the recombinant plasmid using the PEG-calcium method [39]. Briefly, 10 μg plasmid DNA was mixed with 100 μL protoplasts (4 × 10^5^ cells/mL), followed by gently combined with PEG transfection solution (40% w/v PEG 4000, 0.6 mM mannitol, 100 mM CaCl₂), and then incubated at 25 °C in the dark for 15 min. After washing with 440 μL W5 solution (150 mM NaCl, 100 mM CaCl₂, 0.5 mM KH₂PO₄, 0.5 mM MES, pH 4.8), the protoplasts were incubated overnight with W5 medium supplemented with 0.1% (w/v) glucose at 25 °C in the dark [40]. GFP fluorescence was detected using a confocal laser scanning microscope (LSM710; ZEISS, Oberkochen, Germany).

### Transcriptional activation assay of CtDREB1B

For transcriptional activation assay, the full-length *CtDREB1B* CDS was cloned into the pGBKT7 (BD) and PGBKT7-VP16 (BD-VP16) vectors (Pyeast, Wuhan, China) between the EcoR I/BamH I and BamH I/Pst I restriction sites, respectively, to generate recombinant vectors BD-CtDREB1B and BD-VP16-CtDREB1B. The recombinant plasmids and the empty vectors were transformed into yeast strain Y2HGold and cultured on SD/-Trp medium at 30 ℃ for 2–3 days. Then single fresh colonies were selected, resuspended in ddH₂O, and adjusted to an OD₆₀₀ of 0.3. Following 10-fold and 100-fold serial dilutions, 3 μL of each suspension was spotted onto SD/-Trp, SD/-His/-Trp + X-α-gal, and SD/-Ade/-His/-Trp + X-α-gal selective plates. All plates were incubated at 30 °C for 3–5 days, and the growth status of each sample was recorded to evaluate the transcriptional repression activity of CtDREB1B.

### Stress tolerance assays of *CtDREB1B* in yeast

The CDS of *CtDREB1B* was amplified using gene-specific primers containing homologous arms and inserted into the pYES2 vector between the EcoRI and XhoI restriction sites. The recombinant plasmid pYES2::CtDREB1B and empty pYES2 plasmid were heat-transformed into yeast strain BY4741 on SD/-Ura medium at 30℃ for 2–3 days. Then single colonies were picked and resuspended in 100 μL sterile ddH₂O, and the OD₆₀₀ was normalized. Cultures were serially diluted to concentrations of 1.0, 10⁻^1^, 10⁻^2^, and 10⁻^3^ with sterile ddH₂O. Then 3 μL of each dilution was spotted onto SG-Ura (SGR) plates containing either 0, 2, or 4 M NaCl, 0.5 or 1.5 M mannitol, followed by incubation at 30 °C for 3–5 days. Differences in colony growth were observed and recorded.

### Genetic transformation of *A. thaliana*

The *CtDREB1B* CDS was amplified and ligated into the pSuper1300 vector driven by the CaMV35S promoter between XbaI and SalI restriction sites. The resulting plasmid was introduced into *Agrobacterium tumefaciens* GV3101 and transformed into *A. thaliana* Columbia-0 (WT) plants using the floral-dip method [41]. Transgenic *A. thaliana* plants were screened on 1/2 MS solid medium supplemented with 30 μg·mL^−1^ HygB and further confirmed by PCR using hyg-F/R primers (Table S1). Homozygous T3 transgenic lines (OE-3, OE-4, OE-16) were used for subsequent analyses.

### Tolerance of *CtDREB1B* transgenic *A. thaliana* to drought and salt stress treatments

T3 homozygous transgenic lines (OE-3, OE-4, OE-16) and WT *A. thaliana* seeds were placed at 4 °C for 3 days. After vernalization, seeds were evenly sown in commercial substrate (Pindstrup Substrste No.4, Shanghai, China) and cultured in a growth chamber for 7 days under the following conditions: temperature 23 °C, relative humidity 50%, light intensity 10 000 lx, and a 16 h light/8 h dark photoperiod. Subsequently, uniformly growing seedlings were transferred into pots containing equal amounts of substrate and maintained under well-watered conditions for two additional weeks. Prior to stress induction, all plants were saturated with water, and each pot was weighed to standardize initial soil moisture. Drought and salt stress treatments began 4 days after water withholding. For drought stress, plants were treated by withholding irrigation, while salt-stressed plants were irrigated three times with 300 mM NaCl over a 7 days period. Control plants were watered every 3 days. Throughout the experiment, pots were rotated daily to minimize positional effects. On the 7th day of treatment, when phenotypic differences were most significant, phenotypes were photographed, and fresh leaves were sampled for DAB and NBT staining. Leaf samples were simultaneously collected, immediately frozen in liquid nitrogen, and stored at − 80 °C for subsequent physiological index measurements and RNA extraction to analyze the relative expression levels of related genes. Each treatment was performed with three independent biological replicates, and each replicate contained at least 12 plants.

### NBT and DAB staining

Histochemical staining with NitroBlue Tetrazolium (NBT) and 3,3-diaminobenzidine (DAB) was used to detect superoxide anion radicals (O₂⁻) and hydrogen peroxide (H₂O₂) accumulation in *A. thaliana* leaves under stress, respectively. Staining was performed as previously described with minor modifications [42]. Leaves from the same position were soaked in 1 mg·ml^−1^ NBT solution (pH = 7.5) or a 1 mg·ml^−1^ DAB solution (pH = 3.8) and incubated at room temperature in the dark for 24 h. After staining, leaves were decolorized by boiling in 95% ethanol for 15 min and photographed with a digital camera.

### Physiological index determination and stress-responsive gene expression analysis

The frozen leaf samples were ground into powder in liquid nitrogen. The powder was accurately weighed and mixed with 9 volumes of 0.1 M PBS (pH7 ~ 7.4) (weight(g):volume(mL) = 1:9), vortexed for 1 min, and centrifuged at 4000 rpm for 10 min. After which the supernatant was used for the determination of SOD, POD, and CAT activities using commercial kits (SOD: A001-3; POD: A084-3–1; CAT: A007-1–1; Nanjing Jiancheng Bioengineering Institute, Nanjing, China) according to the manufacturer's instructions. Relative expression levels of classic stress-responsive genes in transgenic and WT plants under drought and salt stress were analyzed by qRT-PCR as described avove. Primers used here are listed in Table S1.

### Statistical analysis

Statistically significant differences (*p* < 0.05) in gene expression levels and physiological indices between transgenic and WT plants were analyzed using one-way analysis of variance (ANOVA), and followed by Tukey's Honestly Significant Difference (Tukey HSD) test for post hoc multiple comparisons using SPSS 20. All measurements were performed with at least three independent biological replicates, and data are presented as the mean ± standard deviation (SD).

## Results

### Sequence analysis of *CtDREB1B*

**Fig. 1:**
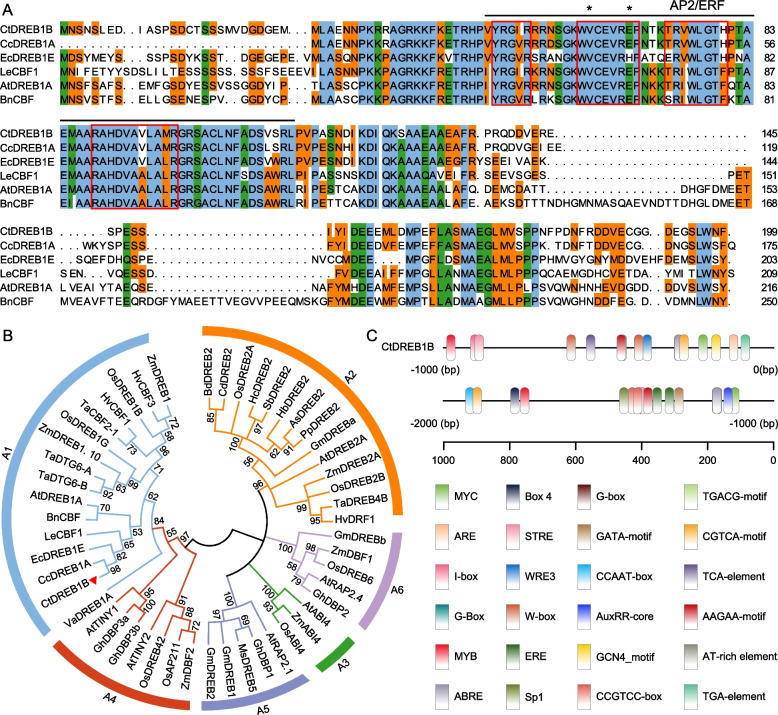
Sequence analysis of *CtDREB1B*. **A** Multiple sequence alignment of CtDREB1B and its homologs from various plant species. The black line indicates the conserved DNA-binding domain (AP2/ERF domain). Four red boxes represent three β-sheets and one α-helix, respectively. Asterisks indicate residues V14 and E19. **B** Phylogenetic tree of CtDREB1B and DREB proteins from other plant species. The accession numbers for genes from other species are listed in Table S2. **C** Promoter analysis of *CtDREB1B*

Given the definite role of *Arabidopsis AtDREB1A* in abiotic stress adaptation, we performed a BLAST search using the AtDREB1A sequence to mine homologous genes in *Carthamus tinctorius*. CtDREB1B shared high sequence identity with AtDREB1A and was therefore selected for further systematic analysis. The *CtDREB1B* gene contains a 600 bp open reading frame, encoding a putative polypeptide of 199 amino acids. Multiple sequence alignment revealed that CtDREB1B possesses a typical AP2/ERF domain, which is composed of three antiparallel β-sheets and one α-helix. Sequence identity analysis indicated that CtDREB1B exhibits high conservation within the AP2 domain when compared with five other DREB proteins. Except for EcDREB1E, all five DREB members contain the highly conserved amino acid residues V14 and E19, which are signature residues of the DREB family (Fig. 1A). These results confirm that the gene isolated in this study belongs to the DREB subfamily.

Phylogenetic analysis was performed on AP2/ERF proteins including CtDREB1B and other plant AP2/ERF proteins. According to the classification of AP2/ERF proteins in *A. thaliana* [6], all these sequences were clustered into six subgroups according to their AP2 domains characteristics (Fig. 1B). As shown in Fig. 1B, CtDREB1B was grouped with CcDREB1A, with which it showed the closest phylogenetic relationship, and was classified into the A1 subgroup together with 15 other DREB proteins (Fig. 1B).

Promoters are pivotal regulators of gene expression, and dissecting their cis-acting elements provides insights into the potential regulatory networks of target genes. To elucidate the regulatory mechanism of *CtDREB1B*, a 2 000 bp sequence upstream of its ATG start codon was retrieved and analyzed using the PlantCARE database. As shown in Fig. 1C, the *CtDREB1B* promoter contains four major categories of cis-acting elements. The first category was plant hormone-responsive elements, including seven types: CGTCA-motif, TGACG-motif, ABRE, TCA-element, AuxRR-core, TGA-element, and AAGAA-motif, among these, ABRE was the most abundant. The second category was light-responsive elements, five types were detected, namely G-Box, Sp1, I-box, Box 4, and GATA-motif, with G-Box being the most abundant. The third category was stress-related elements, eight types were identified, including: ARE, W-box, STRE, ERE, WRE3, MYC, CCAAT-box, and MYB, notably, MYB elements were particularly enriched. The fourth category was plant growth and development-related elements, including GCN4-motif, AT-rich element, and CCGTCC box (Fig. 1C). This suggests that *CtDREB1B* may be integrated into complex regulatory networks to mediate plant responses to environmental cues and coordinate growth and development.

### Expression pattern analysis of *CtDREB1B*

**Fig. 2:**
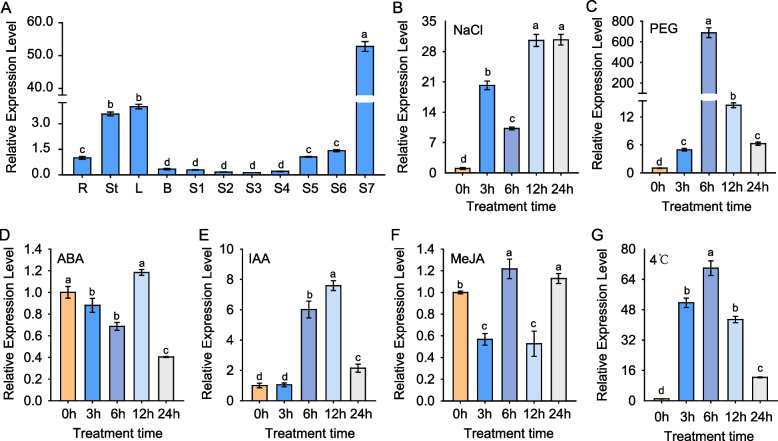
Expression pattern analysis of *CtDREB1B* in safflower. **A** Expression patterns of *CtDREB1B* in various safflower tissues; (**B**–**G**) Expression patterns of *CtDREB1B* under high salt, drought, ABA, IAA, MeJA, and low-temperature treatments, respectively. For each treatment, the expression level at 0 h was used as the control for normalization. All experiments were performed in three biological replicates. Data bars represent the mean ± standard deviation (SD) of three replicates. Statistical analysis was performed using one-way ANOVA, and different lowercase letters indicate significant differences (*p* < 0.05)

The expression pattern of a gene is often closely related to its function. Therefore, qRT-PCR was performed to examine the expression level of *CtDREB1B* in various tissues (root, stem, leaf, bract) and at seven different flowering stages (S1–S7) in safflower. The results showed that *CtDREB1B* exhibited the highest expression level at the S7 flowering stage, followed by leaves and stems, whereas relatively low expression was detected in bracts and during early flowering stages, indicating an obvious tissue- and stage-specific expression pattern (Fig. 2A).

Numerous studies have demonstrated that *DREB* genes are widely involved in plant responses to various abiotic stresses. To determine whether *CtDREB1B* participates in stress and hormone signaling pathways, its transcript levels were analyzed in safflower leaves under salt, drought, ABA, IAA, MeJA, and low-temperature treatments.

The results showed that under salt stress, *CtDREB1B* expression was rapidly induced within 3 h and peaked at 24 h, showing a 30.7-fold increase compared with the control (Fig. 2B). Under drought stress, *CtDREB1B* was significantly up-regulated and reached a maximum at 6 h, with an approximately 687.7-fold induction relative to the control (Fig. 2C). In contrast, exposure to ABA or MeJA treatments, *CtDREB1B* transcript levels first decreased significantly and then gradually returned to near control levels (Fig. 2D and F). Under IAA treatment, *CtDREB1B* expression was significantly enhanced and peaked at 12 h, showing a 7.6-fold increase (Fig. 2E). Under cold stress, *CtDREB1B* was strongly induced at all time points and reached the highest level at 6 h, with a 69.9-fold increase compared with the control (Fig. 2G).

Taken together, these results demonstrate that *CtDREB1B* expression in safflower leaves is highly responsive to salt, drought, and cold stresses, implying that *CtDREB1B* may function as an important regulator in plant abiotic stress responses.

### Subcellular localization and transcriptional activation activity analysis of CtDREB1B

**Fig. 3:**
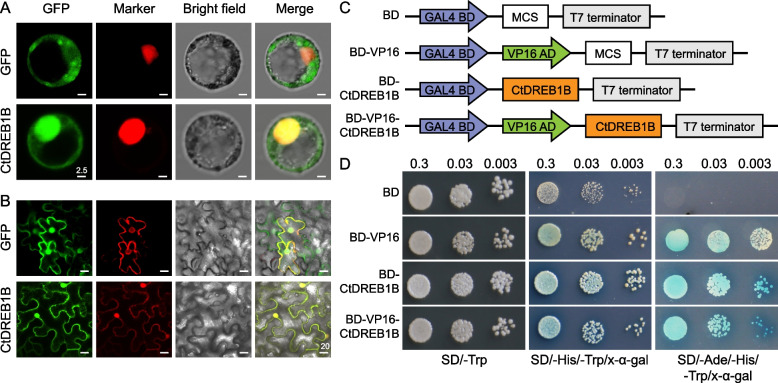
Subcellular localization and transcriptional activation activity analysis of CtDREB1B. **A** Subcellular localization of CtDREB1B in maize leaf protoplasts; (**B**) Subcellular localization of CtDREB1B in tobacco leaf epidermal cells. Maize leaf protoplasts and tobacco leaves were transformed with 35S-GFP (control) or 35S-CtDREB1B-GFP (fusion construct) via *Agrobacterium* infection, and observed and photographed under a laser confocal microscope at 16 h and 60 h, respectively. From left to right: GFP fluorescence, marker fluorescence, bright-field image of cell morphology, and merged image. **C** Schematic diagram of the vectors used for transcriptional activation assay; (**D**) Transcriptional activation activity analysis of CtDREB1B in yeast. The fusion protein of GAL4 DNA-binding domain and CtDREB1B was expressed in yeast strain Y2HGold. The BD vector was used as the negative control, and the full-length *CtDREB1B* was fused to the GAL4 BD domain. Transformed yeast cells containing BD (negative control), BD-VP16 (positive control), BD-CtDREB1B, and BD-VP16-CtDREB1B were cultured on SD/-Trp, SD/-His/-Trp + X-α-gal, and SD/-Ade/-His/-Trp + X-α-gal media. X-α-gal staining was performed to detect β-galactosidase activity

To investigate the biological function of *CtDREB1B*, its subcellular localization was first determined. A recombinant plasmid was constructed by fusing the *CtDREB1B* coding sequence with green fluorescent protein (GFP) under the control of the CaMV 35S promoter. The resulting 35S::CtDREB1B-GFP fusion construct was then transformed into maize leaf protoplasts and tobacco leaf epidermal cells via *Agrobacterium*-mediated transformation, respectively. For subcellular localization analysis, maize protoplasts were only co-transformed with the nuclear marker H2B-mCherry, while tobacco epidermal cells were co-infiltrated with both nuclear and plasma membrane markers. As shown in Fig. 3A and B, in the control group, the green fluorescence signal was widely distributed throughout the entire cell in both materials. In comparison, the fluorescence signal of the 35S::CtDREB1B-GFP fusion protein specifically overlapped with the nuclear marker. Moreover, in tobacco cells, the CtDREB1B-GFP signal also overlapped with the plasma membrane marker. These results collectively demonstrated that CtDREB1B localizes specifically to both the nucleus and cell membrane.

To examine whether CtDREB1B exhibits transcriptional activation activity, the recombinant vectors BD-CtDREB1B and BD-VP16-CtDREB1B were generated (Fig. 3C). The confirmed fusion constructs and empty vectors were separately transformed into yeast Y2HGold competent cells. The results showed that all transformants grew well on SD/-Trp medium. In contrast, only the positive control (BD-VP16), BD-CtDREB1B, and BD-VP16-CtDREB1B yeast strains could grow normally on SD/-His/-Trp + X-α-gal and SD/-Ade/-His/-Trp + X-α-gal media, and the colonies turned blue. Notably, yeast colonies harboring BD-CtDREB1B and BD-VP16-CtDREB1B showed a darker blue color, indicating that CtDREB1B possesses strong transcriptional activation activity in yeast (Fig. 3D).

### *CtDREB1B* enhances salt and drought tolerance in yeast

**Fig. 4:**
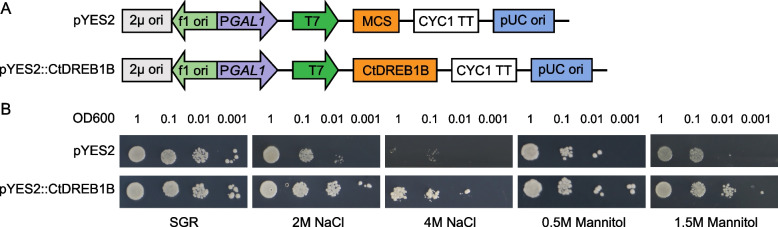
Functional analysis of *CtDREB1B* in yeast grown under drought and salt stresses. **A** Schematic diagrams of pYES2 and pYES2-CtDREB1B vectors. **B** Yeast growth on SGR medium supplemented with different concentrations of NaCl (0, 2, and 4 M) or mannitol (0, 0.5, and 1.5 M) for 5 days

To assess the potential function of *CtDREB1B* under abiotic stress, yeast cells transformed with empty vector pYES2 or recombinant vector pYES2-CtDREB1B were grown on SGR medium supplemented with various concentrations of NaCl (0, 2, and 4 M) or mannitol (0, 0.5, and 1.5 M) for 3–5 days. The results showed that the growth of both transgenic and control yeast cells was gradually inhibited with increasing concentrations of NaCl or mannitol. However, yeast cells expressing *CtDREB1B* displayed significantly enhanced tolerance to salt and osmotic stress compared with the control, especially under 4 M NaCl and 1.5 M mannitol treatments (Fig. 4). These results indicate that *CtDREB1B* enhances the tolerance of yeast cells to salt and drought stresses.

### *CtDREB1B* enhances salt and drought tolerance in *A. thaliana*

**Fig. 5:**
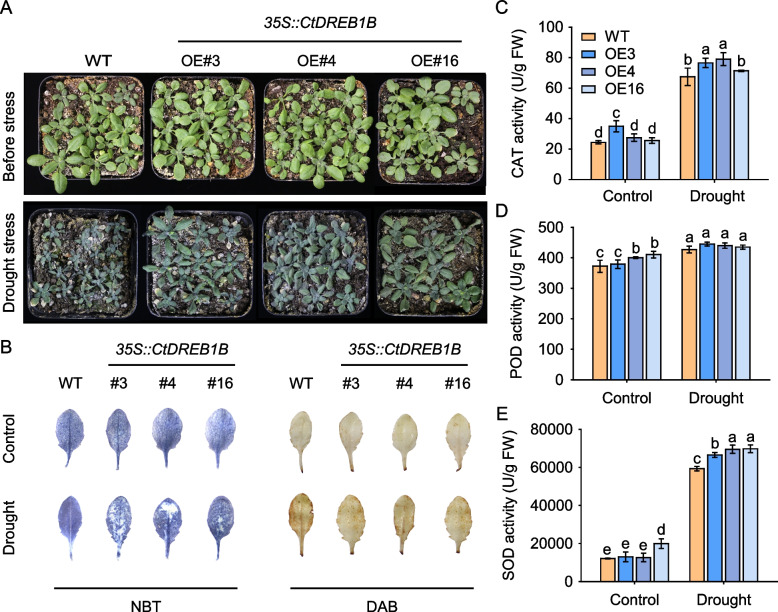
Overexpression of *CtDREB1B* enhances drought stress resistance in *A. thaliana*. **A** Phenotypes of wild-type (WT) and *CtDREB1B*-overexpressing plants after drought treatment; (**B**) DAB and NBT staining of WT and *CtDREB1B*-overexpressing plants under control and drought conditions; (**C**–**E**) CAT, POD, and SOD activities in WT and *CtDREB1B*-overexpressing plants under control and drought conditions. Data bars represent the mean ± standard deviation (SD) of three replicates. Statistical analysis was performed using one-way ANOVA. Different lowercase letters indicate significant differences (*p* < 0.05)

To further explore the function of *CtDREB1B* in plant abiotic stress responses, wild-type and *CtDREB1B*-overexpressing *A. thaliana* plants were subjected to drought and salt treatments. After 5 days of water deprivation, transgenic plants overexpressing *CtDREB1B* exhibited visibly stronger drought tolerance than wild-type plants (Fig. 5A). Meanwhile, DAB and NBT staining showed weaker signal intensities in transgenic lines than in wild type, indicating lower accumulation of H_2_O_2_ and O_2_^−^ (Fig. 5B).

**Fig. 6:**
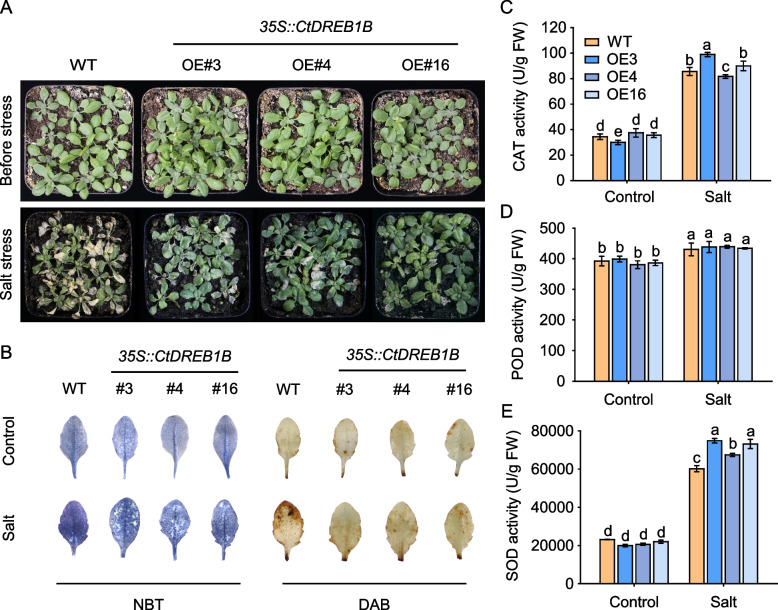
Overexpression of *CtDREB1B* enhances salt stress resistance in *A. thaliana*. **A** Phenotypes of wild-type (WT) and *CtDREB1B*-overexpressing plants after salt treatment; (**B**) DAB and NBT staining of WT and *CtDREB1B*-overexpressing plants under control and salt conditions; (**C**–**E**) CAT, POD, and SOD activities in WT and *CtDREB1B*-overexpressing plants under control and salt conditions. Data bars represent the mean ± standard deviation (SD) of three replicates. Statistical analysis was performed using one-way ANOVA. Different lowercase letters indicate significant differences (*p* < 0.05)

For salt tolerance analysis, plants were treated with 300 mM NaCl for 5 days. *CtDREB1B*-overexpressing *A. thaliana* plants showed markedly enhanced salt tolerance compared with wild-type plants (Fig. 6A). Consistent with the drought treatment, DAB and NBT staining intensities were also lower in transgenic plants under salt stress, suggesting reduced ROS accumulation (Fig. 6B). Collectively, these results demonstrate that overexpression of *CtDREB1B* effectively improves drought and salt tolerance in *A. thaliana*.

### Overexpression of *CtDREB1B* affects antioxidant enzyme activities in *A*.* thaliana*

Abiotic stress usually triggers excessive ROS accumulation and oxidative damage in plants. To investigate whether *CtDREB1B* modulates the antioxidant defense system, the activities of SOD, POD, and CAT were measured in wild-type and transgenic *A. thaliana* under drought and salt stresses. The results showed that under drought stress, CAT and SOD activities were significantly higher in *CtDREB1B*-overexpressing plants than in wild-type plants, while POD activity showed no significant difference (Fig. 5C–E). Similarly, under salt stress, CAT and SOD activities were remarkably elevated in *CtDREB1B*-overexpressing plants compared with the wild type, whereas POD activity showed no obvious difference (Fig. 6C–E). These results suggest that *CtDREB1B* enhances drought and salt tolerance in *A. thaliana*, at least partially by regulating antioxidant enzyme activities to reduce ROS levels.

### Overexpression of *CtDREB1B* affects the expression of classic stress-related genes in *A*.* thaliana*

**Fig. 7:**
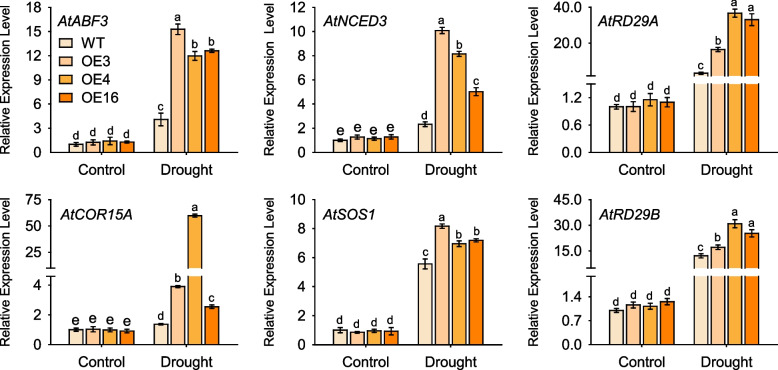
Relative expression levels of classic abiotic stress-responsive genes in *CtDREB1B* transgenic lines compared with wild-type plants under drought stress analyzed by qRT-PCR. All experiments were performed in three replicates. Data are presented as mean ± standard deviation (SD). Statistical analysis was conducted using one-way ANOVA. Different lowercase letters indicate significant differences (*p* < 0.05)

To determine the molecular mechanism by which *CtDREB1B* enhances stress tolerance, the transcript levels of six well-known stress-responsive genes (*AtABF3*, *AtNCED3*, *AtRD29A*, *AtRD29B*, *AtCOR15A*, and *AtSOS1*) were analyzed by qRT-PCR under drought and salt conditions. Under drought stress, all six genes were significantly induced in both wild-type and transgenic plants, with much higher induction levels in *CtDREB1B*-overexpressing lines (Fig. 7). Among them, *AtRD29A*, *AtRD29B*, and *AtCOR15A* exhibited the most dramatic upregulation, reaching 36.7-, 30.9-, and 59.8-fold higher expression than in wild-type plants, respectively.

**Fig. 8:**
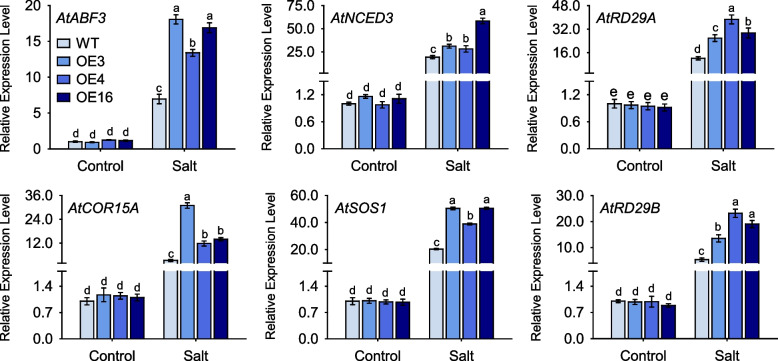
Relative expression levels of classic abiotic stress-responsive genes in *CtDREB1B* transgenic lines compared with wild-type plants under salt stress analyzed by qRT-PCR. All experiments were performed in three replicates. Data are presented as mean ± standard deviation (SD). Statistical analysis was conducted using one-way ANOVA. Different lowercase letters indicate significant differences (*p* < 0.05)

Under salt stress, the expression patterns of these stress-related genes were similar to those under drought stress. Notably, *AtNCED3*, *AtRD29A*, *AtCOR15A*, and *AtSOS1* were strongly activated in transgenic plants, with maximal expression levels 58.5-, 38.6-, 30.9-, and 30.9-fold higher than in wild-type plants, respectively (Fig. 8). These results demonstrate that overexpression of *CtDREB1B* significantly enhances the transcriptional induction of core drought- and salt-responsive genes, further supporting that *CtDREB1B* functions as a positive regulator in plant abiotic stress tolerance.

## Discussion

DREB transcription factors are core regulators in plant responses to drought, salt, and other abiotic stresses. Therefore, the identification and characterization of DREB transcription factors lay a foundation for analyzing the molecular mechanisms of stress tolerance and developing new stress-tolerant crop varieties. With the rapid development of high-throughput sequencing and the continuous enrichment of plant genomic resources, the functions and regulatory mechanisms of DREB proteins have been widely studied in many plant species. However, the function of DREB transcription factors in safflower remains largely unknown. In this study, we cloned the *CtDREB1B* gene from safflower, which contains a highly conserved AP2/ERF domain. Previous studies have shown that valine (V) at position 14 in the AP2 domain is critical for specific DNA-binding activity, while glutamate (E) at position 19 shows relatively minor variation [6]. In the present study, sequence alignment revealed that the 14th amino acid was uniformly valine (V) among all compared sequences, and all contained glutamate (E) at position 19 except for EcDREB1E. These results are consistent with previous reports and further confirm that *CtDREB1B* belongs to the DREB subfamily.

Most characterized DREB proteins localize to the nucleus to activate transcription. Interestingly, subcellular localization assays in both tobacco epidermal cells and maize protoplasts showed that CtDREB1B was distributed in both the nucleus and cell membrane. This dual localization pattern is rare in the DREB family and has only been reported in rice [43]. OsDREB1B localized to both the nucleus and plasma membrane under normal conditions, but translocated exclusively into the nucleus upon cold stress, implying a potential nuclear envelope-anchored sequestration-release mechanism involved in stress responses. Under normal conditions, the protein may be tethered to the membrane in an inactive state. Upon stress stimulation, it may be released into the nucleus to bind DRE/CRT cis-elements and rapidly activate downstream stress-responsive genes. This potential regulatory strategy may enable plants to mount rapid and flexible stress responses without the growth penalty caused by constitutive nuclear activation, thus balancing stress resistance and growth fitness. Our findings broaden the diversity of subcellular localization patterns and regulatory models within the DREB family and provide new insights into the molecular basis of plant drought and salt tolerance.

Expression pattern analysis revealed that *CtDREB1B* was predominantly expressed in the S7 flower stage and leaves, and was significantly induced by various abiotic stresses, including low temperature, drought, and salt stress, as well as treatment with IAA, but not by ABA and MeJA. This expression profile differs from some previously reported DREB homologs. For instance, *LcDREB3a*, *GhDBP2*, and *GmDREB2* were up-regulated by low temperature, ABA, salt, or osmotic stress [44–46]. By contrast, *AtDREB1A* and *AtDREB2A* were insensitive to ABA [6]. In rice, *OsDREB1A* and *OsDREB1B* were rapidly induced by cold but did not respond to ABA, while *OsDREB2A* was induced within 24 h after dehydration and salt stress, and exhibited only weak responses to ABA and cold [21]. The *TaDREB1* isolated from wheat was strongly induced by cold, but showed poor responses to drought, salt, and ABA [47], and *WCBF2* was rapidly induced by low temperature and drought, but not regulated by ABA [48]. Similarly, ABA treatment failed to induce the expression of *HcDREB2*, conversely, *HcDREB2* expression was rapidly induced by low temperature, drought, and high salt [49]. Taken together, these results indicate that the expression responses of *DREB* genes to abiotic stresses vary considerably among different plant species, reflecting diverse evolutionary adaptations and regulatory mechanisms within this transcription factor family.

**Fig. 9:**
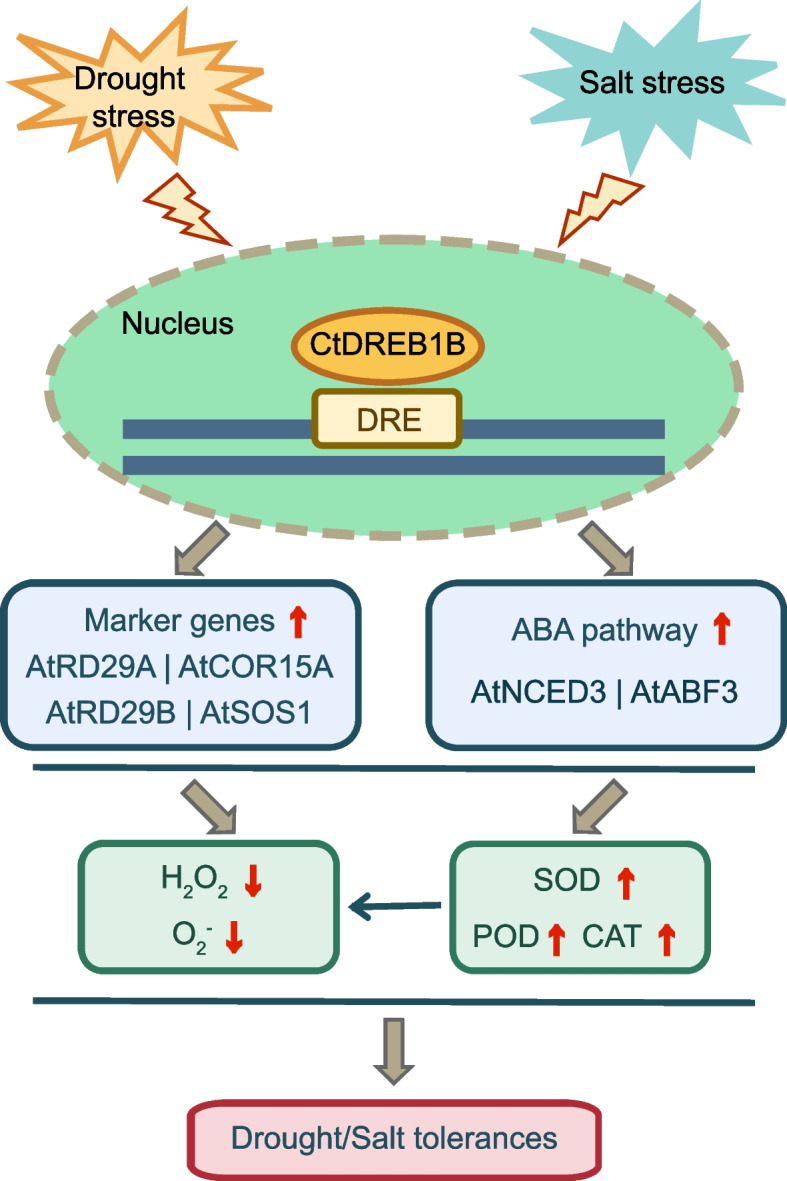
A brief model of the potential mechanism by which *CtDREB1B* enhances drought and salt stress tolerance in *A. thaliana*. When exposed to salt and drought stress, transgenic *A. thaliana* plants overexpressing *CtDREB1B* gene was able to upregulated the expression of typical stress-related and ABA biosynthesis genes, which consequently enhanced the activities of SOD, POD and CAT, decreased the accumulation of H_2_O_2_ and O_2_^−^, and ultimately improved the tolerance of transgenic *A. thaliana* to salt and drought stress

In this study, heterologous expression of *CtDREB1B* in yeast improved the tolerance of yeast strains to drought and salt stress to a certain extent. Moreover, transgenic *A. thaliana* plants overexpressing *CtDREB1B* also exhibited enhanced tolerance to drought and salt stress, indicating that *CtDREB1B* may act as a positive regulator involved in plant abiotic stress tolerance. Abiotic stress usually triggers excessive accumulation of ROS, such as H₂O₂ and O₂⁻, leading to severe oxidative damage [50, 51], and plants maintain ROS homeostasis mainly through the antioxidant enzyme system. In our study, transgenic plants overexpressing *CtDREB1B* showed significantly reduced accumulation of H₂O₂ and O₂⁻, accompanied by increased activities of SOD, POD, and CAT. These results suggest that *CtDREB1B* may enhance drought and salt tolerance, at least in part, by activating the antioxidant system and alleviating ROS-mediated oxidative damage. DREB transcription factors typically improve plant tolerance to multiple abiotic stresses by binding to DRE/CRT cis-elements and activating the expression of downstream stress-responsive genes [9, 52]. Therefore, we analyzed the relative expression levels of representative stress-responsive genes in transgenic and wild-type *A. thaliana*. qRT-PCR analysis showed that the transcript levels of stress marker genes, including *AtRD29A*, *AtRD29B*, *AtCOR15A*, *AtABF3*, *AtNCED3*, and *AtSOS1*, were significantly upregulated in transgenic *A. thaliana* compared with the wild type. These findings are consistent with observations in *StDREB2*-overexpressing cotton [53] and *BaDBL1*-overexpressing *A. thaliana* [54], where enhanced antioxidant enzyme activities and upregulated stress-related gene expression led to reduced ROS accumulation and improved stress tolerance. Notably, the expression of *CtDREB1B* was strongly induced by drought and salt stress, but showed no significant response to exogenous ABA treatment. As a conserved DREB1/CBF-type transcription factor, this expression pattern indicates that the transcriptional regulation of *CtDREB1B* belongs to the ABA-independent pathway and it acts upstream of the core ABA signaling cascade [55]. Conversely, ectopic overexpression of *CtDREB1B* significantly upregulated the expression of *AtNCED3*, a rate-limiting gene essential for ABA biosynthesis, as well as *AtABF3* (a core transcription factor in ABA signaling) and the ABA-responsive marker gene *AtRD29B*. These findings illustrate that *CtDREB1B* enhances drought and salt tolerance primarily by activating ABA biosynthesis and reinforcing ABA signal transduction, representing a typical crosstalk between ABA-independent transcriptional induction and ABA-dependent downstream physiological outputs in plant stress responses [56]. In addition, *CtDREB1B* may directly or indirectly upregulate *AtSOS1* to maintain Na⁺ homeostasis under salt stress. Based on these results, we propose a putative regulatory model: stress-induced *CtDREB1B* may activate DRE-containing target genes, stimulate ABA biosynthesis and signaling, enhance the antioxidant system, and maintain ion homeostasis, thereby synergistically improving plant drought and salt tolerance (Fig. 9).

This study provides preliminary characterization of the *CtDREB1B* gene from safflower and its function in heterologous systems, but several limitations should be acknowledged, which also offer valuable insights for future research. First, due to the incomplete establishment of safflower’s transgenic system, all functional verifications of *CtDREB1B* were conducted exclusively in heterologous hosts (yeast and *A. thaliana*), with no in vivo functional verification performed in its native species (safflower itself). Second, the molecular mechanism underlying the dual subcellular localization of CtDREB1B (nucleus and cell membrane) remains elusive. While this study successfully observed the localization pattern, but did not identify the key amino acid sequences or structural domains responsible for membrane anchoring, furthermore, the potential stress-induced nuclear translocation process of CtDREB1B and its regulatory triggers were not thoroughly verified. Third, the direct target genes of *CtDREB1B* have not been identified. Although we observed the upregulation of multiple stress-responsive genes in transgenic *A. thaliana*, these genes may be indirect downstream targets, and the specific DRE/CRT cis-elements bound by *CtDREB1B* and its direct regulatory targets in the stress response pathway remain to be confirmed through complementary techniques such as chromatin immunoprecipitation (ChIP), yeast one-hybrid, and dual-luciferase reporter assays. Future studies will focus on elucidating the precise molecular mechanism underlying the stress-induced release and nuclear translocation of *CtDREB1B*, identifying its direct target genes and uncovering its interacting proteins within the safflower stress response network.

## Conclusions

In this study, we successfully cloned the *CtDREB1B* gene from safflower, which contains a 600 bp full-length CDS encoding a 199-amino-acid protein belonging to the DREB A1 subgroup. The expression of *CtDREB1B* is induced by cold, drought, salt stresses and IAA treatment, but not by ABA and MeJA. CtDREB1B is dual-localized to the nucleus and cell membrane, and has transcriptional activation activity in yeast. Overexpression of *CtDREB1B* in yeast improved the drought and salt tolerance of the strains. Similarly, overexpression of this gene in *A*. *thaliana* led to reduced accumulation of H_2_O_2_ and O_2_^−^, increased activities of SOD, POD and CAT, and upregulated expression of key stress-responsive genes (e.g., *AtRD29A*, *AtRD29B*, *AtCOR15A*, *AtABF3*, *AtNCED3*, and *AtSOS1*). Collectively, our findings uncover a novel regulatory mechanism of drought and salt tolerance mediated by the dual-localized (nucleus and cell membrane) DREB transcription factor CtDREB1B, and expand the current understanding of the functional and subcellular localization diversity of DREB transcription factors. This study also provides a promising candidate gene for the genetic improvement of crop stress tolerance, but further in-depth research is required before its practical application in molecular breeding.

## Supplementary Information

Supplementary Material 1.

Supplementary Material 2.

Supplementary Material 3.

## Data Availability

All data was included in the manuscript and its supplementary materials.

## Abbreviations

DREB: Dehydration-responsive element binding
AP2/ERF: APETALA2/Ethylene-Responsive Factor
RAV(ABI3/VP1): Related to ABI3/VP1
ABI3: Abscisic Acid Insensitive 3
VP1: Viviparous 1
CBF: C-Repeat Binding Factor
NCBI: National Center for Biotechnology Information
DRE/DRE: Dehydration-Responsive Element/C-Repeat
V: Valine
E: Glutamic acid
A: Alanine
D: Aspartic acid
*A. thaliana*: *Arabidopsis thaliana*
ABA: Abscisic Acid
FAD: Fatty Acid Desaturase
PEG: Polyethylene Glycol
MeJA: Methyl Jasmonate
IAA: Indole-3-Acetic Acid
CDS: Coding Sequence
PCR: Polymerase Chain Reaction
qRT-PCR: Quantitative Real-Time PCR
MES: 2-(N-Morpholino) ethanesulfonic acid
WT: Wild Type
NBT: NitroBlue Tetrazolium
DAB: 3,3-Diaminobenzidine
O₂⁻: Superoxide anion radicals
H₂O₂: Hydrogen peroxide
PBS: Phosphate Buffered Saline
SOD: Superoxide dismutase
POD: Peroxidase
CAT: Catalase
ABRE: ABA-Responsive Element
ROS: Reactive Oxygen Species

## References

[1] Mizoi J, Shinozaki K, Yamaguchi-Shinozaki K. AP2/ERF family transcription factors in plant abiotic stress responses. Biochim Biophys Acta. 2012;1819(2):86–96. 10.1016/j.bbagrm.2011.08.004. 21867785 10.1016/j.bbagrm.2011.08.004

[2] Elliott RC, Betzner AS, Huttner E, Oakes MP, Tucker WQ, Gerentes D, et al. AINTEGUMENTA, an APETALA2-like gene of *Arabidopsis* with pleiotropic roles in ovule development and floral organ growth. Plant Cell Online. 1996;8(2):155–68. 10.1105/tpc.8.2.155. 10.1105/tpc.8.2.155 PMC161088 8742707

[3] Gutterson N, Reuber TL. Regulation of disease resistance pathways by AP2/ERF transcription factors. Curr Opin Plant Biol. 2004;7(4):465–71. 10.1016/j.pbi.2004.04.007. 15231271 10.1016/j.pbi.2004.04.007

[4] B UK, N M, P K, K A, S S, P K, et al. Genetic diversity of safflower (*Carthamus tinctorius* L.) germplasm as revealed by SSR markers. Plant Genet Resour. 2017;15(1):1–11. 10.1017/S1479262115000295.

[5] Liu Q, Kasuga M, Sakuma Y, Abe H, Miura S, Yamaguchi-Shinozaki K, et al. Two transcription factors, DREB1 and DREB2, with an EREBP/AP2 DNA binding domain separate two cellular signal transduction pathways in drought-and low temperature-responsive gene expression, respectively, in *Arabidopsis*. Plant Cell. 1998;10(8):1391–406. 10.1105/tpc.10.8.1391. 9707537 10.1105/tpc.10.8.1391 PMC144379

[6] Sakuma Y, Liu Q, Dubouzet JG, Abe H, Shinozaki K, Yamaguchi-Shinozaki K. DNA-binding specificity of the ERF/AP2 domain of *Arabidopsis* DREBs, transcription factors involved in dehydration- and cold-inducible gene expression. Biochem Biophys Res Commun. 2002;290(3):998–1009. 10.1006/bbrc.2001.6299. 11798174 10.1006/bbrc.2001.6299

[7] Nakano T, Suzuki K, Fujimura T, Shin H. Genome-wide analysis of the ERF gene family in *Arabidopsis* and rice. Plant Physiol. 2006;140(2):411–32. 10.1104/pp.105.073783. 16407444 10.1104/pp.105.073783 PMC1361313

[8] Stockinger EJ, Gilmour SJ, Thomashow MF. *Arabidopsis thaliana* CBF1 encodes an AP2 domain-containing transcriptional activator that binds to the C-repeat/DRE, a cis-acting DNA regulatory element that stimulates transcription in response to low temperature and water deficit. Proc Natl Acad Sci U S A. 1997;94(3):1035–40. 10.1073/pnas.94.3.1035. 9023378 10.1073/pnas.94.3.1035 PMC19635

[9] Agarwal PK, Agarwal P, Reddy MK, Sopory SK. Role of DREB transcription factors in abiotic and biotic stress tolerance in plants. Plant Cell Rep. 2006;25(12):1263–74. 10.1007/s00299-006-0204-8. 16858552 10.1007/s00299-006-0204-8

[10] Lata C, Prasad M. Role of DREBs in regulation of abiotic stress responses in plants. J Exp Bot. 2011;62(14):4731–48. 10.1093/jxb/err210. 21737415 10.1093/jxb/err210

[11] Khan MS. The role of DREB transcription factors in abiotic stress tolerance of plants. Biotechnol Biotechnol Equip. 2011;25(3):2433–42. 10.5504/bbeq.2011.0072.

[12] Tang MJ, Lü SY, Jing YX, Zhou XJ, Sun JW, Shen SH. Isolation and identification of a cold-inducible gene encoding a putative DRE-binding transcription factor from *Festuca arundinacea*. Plant Physiol Biochem. 2005;43(3):233–9. 10.1016/j.plaphy.2005.01.015. 15854831 10.1016/j.plaphy.2005.01.015

[13] Wang Y, Wang Y, Meng Z, Wei Y, Du X, Liang C, et al. Elevation of *GhDREB1B* transcription by a copy number variant significantly improves chilling tolerance in cotton. Planta. 2021;254(2):42. 10.1007/s00425-021-03686-1. 34331139 10.1007/s00425-021-03686-1

[14] Shan D, Huang J, Yang Y, Guo Y, Wu C, Yang G, et al. Cotton *GhDREB1* increases plant tolerance to low temperature and is negatively regulated by gibberellic acid. New Phytol. 2007;176(1):70–81. 10.1111/j.1469-8137.2007.02160.x. 17803642 10.1111/j.1469-8137.2007.02160.x

[15] Zhang Y, Chen C, Jin XF, Xiong AS, Peng RH, Hong YH, et al. Expression of a rice *DREB1* gene, *OsDREB1D*, enhances cold and high-salt tolerance in transgenic *Arabidopsis*. BMB Rep. 2009;42(8):486–92. 10.5483/BMBRep.2009.42.8.486. 19712584 10.5483/bmbrep.2009.42.8.486

[16] Cui M, Zhang W, Zhang Q, Xu Z, Zhu Z, Duan F, et al. Induced over-expression of the transcription factor OsDREB2A improves drought tolerance in rice. Plant Physiol Biochem. 2011;49(12):1384–91. 10.1016/j.plaphy.2011.09.012. 22078375 10.1016/j.plaphy.2011.09.012

[17] Zhang XX, Tang YJ, Ma QB, Yang CY, Mu YH, Suo HC, et al. OsDREB2A, a rice transcription factor, significantly affects salt tolerance in transgenic soybean. PLoS ONE. 2013;8(12):e83011. 10.1371/journal.pone.0083011. 24376625 10.1371/journal.pone.0083011 PMC3869746

[18] Agarwal P, Agarwal PK, Joshi AJ, Sopory SK, Reddy MK. Overexpression of PgDREB2A transcription factor enhances abiotic stress tolerance and activates downstream stress-responsive genes. Mol Biol Rep. 2010;37(2):1125–35. 10.1007/s11033-009-9885-8. 19826914 10.1007/s11033-009-9885-8

[19] Chen H, Liu L, Wang L, Wang S, Cheng X. VrDREB2A, a DREB-binding transcription factor from *Vigna radiata*, increased drought and high-salt tolerance in transgenic *Arabidopsis thaliana*. J Plant Res. 2016;129(2):263–73. 10.1007/s10265-015-0773-0. 26646381 10.1007/s10265-015-0773-0

[20] Zhou ML, Ma JT, Zhao YM, Wei YH, Tang YX, Wu YM. Improvement of drought and salt tolerance in *Arabidopsis* and *Lotus corniculatus* by overexpression of a novel DREB transcription factor from *Populus euphratica*. Gene. 2012;506(1):10–7. 10.1016/j.gene.2012.06.089. 22771912 10.1016/j.gene.2012.06.089

[21] Dubouzet JG, Sakuma Y, Ito Y, Kasuga M, Dubouzet EG, Miura S. *OsDREB* genes in rice, *Oryza sativa L.* encode transcription activators that function in drought, high salt and cold responsive gene expression. Plant J. 2003;33(4):751–63. 10.1046/j.1365-313X.2003.01661.x. 12609047 10.1046/j.1365-313x.2003.01661.x

[22] Li T, Huang Y, Khadr A, Wang YH, Xu ZS, Xiong AS. DcDREB1A, a DREB- binding transcription factor from *Daucus carota*, enhances drought tolerance in transgenic *Arabidopsis thaliana* and modulates lignin levels by regulating lignin-biosynthesis- related genes. Environ Exp Bot. 2020;169(0):103896. 10.1016/j.envexpbot.2019.103896.

[23] Xu Y, Hu W, Song S, Ye X, Ding Z, Liu J, et al. MaDREB1F confers cold and drought stress resistance through common regulation of hormone synthesis and protectant metabolite contents in banana. Hortic Res. 2022;10(2):uhac275. 10.1093/hr/uhac275. 36789258 10.1093/hr/uhac275 PMC9923210

[24] Liu BJ, Zhou Y, Lan W, Zhou Q, Li F, Chen F. *LlDREB1G*, a novel DREB subfamily gene from *Lilium longiflorum*, can enhance transgenic *Arabidopsis* tolerance to multiple abiotic stresses. Plant Cell. 2019;138(3):489–506. 10.1007/s11240-019-01644-0.

[25] Sruthy MA, Narayan AJ, Divya P, Syamaladevi P, Appunu C, Chakravarthi M, et al. Overexpression of *EaDREB2* and pyramiding of *EaDREB2* with the pea DNA helicase gene (*PDH45*) enhance drought and salinity tolerance in sugarcane (*Saccharum spp.* hybrid). Plant Cell Rep. 2014;34(2):247–63. 10.1007/s00299-014-1704-6. 25477204 10.1007/s00299-014-1704-6

[26] Yin Y, Jiang X, Ren M, Xue M, Nan D, Wang Z, et al. AmDREB2C, from *Ammopiptanthus mongolicus*, enhances abiotic stress tolerance and regulates fatty acid composition in transgenic *Arabidopsis*. Plant Physiol Biochem. 2018;130(0):517–28. 10.1016/j.plaphy.2018.08.002. 30096686 10.1016/j.plaphy.2018.08.002

[27] Gomashe SS, Ingle KP, Sarap YA, Subramani R, Chand D. Safflower (*Carthamus tinctorius* L.): an underutilized crop with potential medicinal values. Ann Phytomed. 2021;10(1):242–8. 10.21276/ap.2021.10.1.26.

[28] Ren CX, Wu YY, Tang XH, Hu J, Chen J, Wu QH, et al. Safflower’s origin and changes of producing areas. Chin J Chin Mater Med. 2017;42(11):2219–22. 10.19540/j.cnki.cjcmm.2017.0101. 10.19540/j.cnki.cjcmm.2017.0101 28822172

[29] Tu YH, Xue YR, Guo DD, Sun LN, Guo ML. Carthami flos: a review of its ethnopharmacology, pharmacology and clinical applications. Rev Bras Farmacogn. 2015;25(5):553–66. 10.1016/j.bjp.2015.06.001.

[30] Wu ZH, Li RT, Sun MH, Hu XL, Xiao MH, Hu ZH, et al. Current advances of *Carthamus tinctorius* L.: a review of its application and molecular regulation of flavonoid biosynthesis. Med Plant Biol. 2024;3(1):1–11. 10.48130/mpb-0024-0005.

[31] Si W, Yang WH, Guo DD, Wu J, Zhang J, Qiu S, et al. Selective ion monitoring of quinochalcone C-glycoside markers for the simultaneous identification of *Carthamus tinctorius* L. in eleven Chinese patent medicines by UHPLC/QTOF MS. J Pharm Biomed Anal. 2016;117:510–21. 10.1016/j.jpba.2015.09.025. 26476296 10.1016/j.jpba.2015.09.025

[32] Hussain MI, Lyra DA, Farooq M. Salt and drought stresses in safflower: a review. Agron Sustain Dev. 2016;36(1):1–31. 10.1007/s13593-015-0344-8.

[33] Zhan P, Zhong ZC, Xiang NY, Qin R, Jiang XB, et al. Identification of *Carthamus tinctorius* NAC gene family and analysis of drought stress response. China J Chin Mater Med. 2022;47(20):5520–9. 10.19540/j.cnkicjcmm.20220514.104. 10.19540/j.cnki.cjcmm.20220514.104 36471968

[34] Zhang HR, He LW, Li QL, Gao ZM. The sowing time effect of *Carthamus tinctorius* on low temperature growth. Res Pract Chin Med. 2016;30(6):1–4. 10.13728/j.1673-6427.2016.06.001.

[35] Lu DD, Wang LN, Yu YL, Li L, Su XY, Sun Y, et al. Genome-wide identification and functional analyses of the TCP gene family in *Carthamus tinctorius* L. Sci Rep. 2025;15:12970. 10.1038/s41598-025-97743-4. 40234668 10.1038/s41598-025-97743-4 PMC12000515

[36] Lescot M, Dehais P, Thijs G, et al. PlantCARE, a database of plant cis-acting regulatory elements and a portal to tools for in silico analysis of promoter sequences. Nucleic Acids Res. 2002;30(1):325–7. 10.1093/nar/30.1.325. 11752327 10.1093/nar/30.1.325 PMC99092

[37] Rao XY, Huang XL, Zhou ZC, Lin X. An improvement of the 2^-delta delta CT^ method for quantitative real-time polymerase chain reaction data analysis. Biostat Bioinform Biomathe. 2013;3(3):71–85. PMC4280562 25558171

[38] Lu DD, Su XY, Sun Y, Li L, Yu YL, Li CM, et al. Genome-wide identification of the DFR gene family in *Lonicera japonica* Thunb. and response to drought and salt stress. Genes. 2025;16(12):1453. 10.3390/genes16121453. 41465127 10.3390/genes16121453 PMC12732910

[39] Zhao YL, Du HW, Wang YK, Wang HL, Yang SY, Li CH, et al. The calcium-dependent protein kinase ZmCDPK7 functions in heat-stress tolerance in maize. J Integr Plant Biol. 2021;63(3):510–27. 10.1111/jipb.13056. 33331695 10.1111/jipb.13056

[40] Zhao YL, Wang YS, Liu HH, Jiang HF, Xue RL, Wang SL, et al. Chloroplast casein kinase 2 enhances maize thermotolerance via sHSP26 phosphorylation and retrograde HSFA3 regulation. Plant Physiol. 2025;199(3):kiaf540. 10.1093/plphys/kiaf540. 41137455 10.1093/plphys/kiaf540

[41] Clough SJ, Bent AF. Floral dip: a simplified method for *Agrobacterium*-mediated transformation of *Arabidopsis thaliana*. Plant J. 1998;16(6):735–43. 10.1046/j.1365-313x.1998.00343.x. 10069079 10.1046/j.1365-313x.1998.00343.x

[42] Zhang Z, Zhang A, Zhang Y, Zhao J, Wang Y, Zhang L, et al. Ectopic expression of *HaPEPC1* from the desert shrub *Haloxylon ammodendron* confers drought stress tolerance in *Arabidopsis thaliana*. Plant Physiol Biochem. 2024;208:108536. 10.1016/j.plaphy.2024.108536. 38507839 10.1016/j.plaphy.2024.108536

[43] Xu WJ. Identification of rice stress-resistant transcription factor OsDREB family and functional analysis of *OsDREB1B* gene. Shenyang: Shenyang Agricultural University. 2025;46. 10.27327/d.cnki.gshnu.2025.001112.

[44] Huang B, Jin LG, Liu JY. Identification and characterization of the novel gene *GhDBP2* encoding a DRE-binding protein from cotton (*Gossypium hirsutum*). J Plant Physiol. 2008;165(2):214–23. 10.1016/j.jplph.2006.11.003. 17224201 10.1016/j.jplph.2006.11.003

[45] Chen M, Wang QY, Cheng XG, Xu ZS, Li LC, Ye XG, et al. GmDREB2, a soybean DRE-binding transcription factor, conferred drought and high-salt tolerance in transgenic plants. Biochem Biophys Res Commun. 2007;353(2):299–305. 10.1016/j.bbrc.2006.12.027. 17178106 10.1016/j.bbrc.2006.12.027

[46] Peng XJ, Ma XY, Fan WH, Su M, Cheng LQ, Alam I, et al. Improved drought and salt tolerance of *Arabidopsis thaliana* by transgenic expression of a novel *DREB* gene from *Leymus chinensis*. Plant Cell Rep. 2011;30(8):1493–502. 10.1007/s00299-011-1058-2. 21509473 10.1007/s00299-011-1058-2

[47] Shen YG, Zhang WK, He SJ, Zhang JS, Liu Q, Chen SY. An EREBP/AP2-type protein in *Triticum aestivum* was a DRE-binding transcription factor induced by cold, dehydration and ABA stress. Theor Appl Genet. 2003;106(5):923–30. 10.1007/s00122-002-1131-x. 12647068 10.1007/s00122-002-1131-x

[48] Kume S, Kobayashi F, Ishibashi M, Ohno R, Nakamura C, Takumi S. Differential and coordinated expression of *Cbf* and *Cor*/*Lea* genes during long-term cold acclimation in two wheat cultivars showing distinct levels of freezing tolerance. Genes Genet Syst. 2005;80(3):185–97. 10.1266/ggs.80.185. 16172531 10.1266/ggs.80.185

[49] Chen YX, Huang LK, Yan HD, Zhang XQ, Xu B, Ma X, et al. Cloning and characterization of an ABA-independent DREB transcription factor gene, *HcDREB2*, in *Hemarthria compressa*. Hereditas. 2016;153(1):3. 10.1186/s41065-016-0008-y. 28096765 10.1186/s41065-016-0008-y PMC5224587

[50] Gill SS, Tuteja N. Reactive oxygen species and antioxidant machinery in abiotic stress tolerance in crop plants. Plant Physiol Biochem. 2010;48(12):909–30. 10.1016/j.plaphy.2010.08.016. 20870416 10.1016/j.plaphy.2010.08.016

[51] Van Breusegem F, Dat JF. Reactive oxygen species in plant cell death. Plant Physiol. 2006;141(2):384–90. 10.1104/pp.106.078295. 16760492 10.1104/pp.106.078295 PMC1475453

[52] Agarwal PK, Jha B. Transcription factors in plants and ABA dependent and independent abiotic stress signalling. Biol Plant. 2010;54(2):201–12. 10.1007/s10535-010-0038-7.

[53] El-Esawi MA, Alayafi AA. Overexpression of *StDREB2* transcription factor enhances drought stress tolerance in cotton (*Gossypium barbadense* L.). Genes. 2019;10(2):142. 10.3390/genes1002142. 30769841 10.3390/genes10020142 PMC6409991

[54] Liang YQ, Li XS, Yang RR, Gao B, Yao JX, Oliver MJ, et al. *BaDBL1*, a unique DREB gene from desiccation tolerant moss *Bryum argenteum*, confers osmotic and salt stress tolerances in transgenic *Arabidopsis*. Plant Sci. 2021;313(Suppl C):111047. 10.1016/j.plantsci.2021.111047. 34763851 10.1016/j.plantsci.2021.111047

[55] Y T, M J, Ys K. ABA-dependent and ABA-independent signaling in response to osmotic stress in plants. Curr Opin Plant Biol. 2014;21:133–9. 10.1016/j.pbi.2014.07.009. 25104049 10.1016/j.pbi.2014.07.009

[56] Xu K, Chen S, Wan Y, Li L. A DREB1 transcription factor from *Ammopiptanthus nanus* enhances drought and salt tolerance by promoting ABA biosynthesis. Plant Sci. 2020;299:110587. 10.1016/j.plantsci.2020.110587.

