# Supplementary figures and images for: Unveiling the role of CtDREB1B from safflower: enhancing plant resistance to drought and salt
Source: BMC Plant Biol. 2026 May 23;26:1223. doi: 10.1186/s12870-026-08996-8 (PMC13378227; doi:10.1186/s12870-026-08996-8)

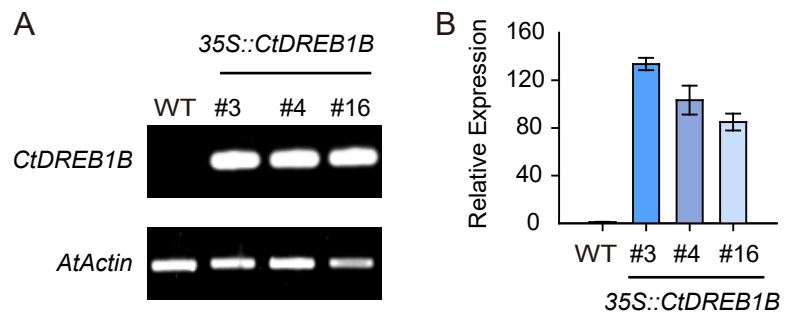

Supplement: Supplementary file 1 — Supplementary Material 1. [file 12870_2026_8996_MOESM1_ESM.pdf]
